# Psychological distress facing the COVID-19 pandemic in dental interns from the Peruvian capital: A cross-sectional study under a multivariable regression model

**DOI:** 10.3389/fpubh.2022.996921

**Published:** 2022-12-21

**Authors:** César Cayo-Rojas, Nancy Córdova-Limaylla, Marysela Ladera-Castañeda, Gissela Briceño-Vergel, Carlos López-Gurreonero, Manuel Castro-Mena, Alberto Cornejo-Pinto, Regina Agramonte-Rosell, Luis Cervantes-Ganoza

**Affiliations:** ^1^School of Stomatology, Universidad Privada San Juan Bautista, Lima, Peru; ^2^Faculty of Dentistry and Postgraduate School, Grupo de Investigación Salud y Bienestar Global, Universidad Nacional Federico Villarreal, Lima, Peru; ^3^School of Stomatology, Universidad Científica del Sur, Lima, Peru; ^4^Faculty of Stomatology, Universidad Inca Garcilaso de la Vega, Lima, Peru

**Keywords:** COVID-19 peritraumatic distress index, dental interns, dentistry, behavioral disorders, Peru, psychological distress

## Abstract

**Aim:**

Psychological distress can be considered a maladaptive response to a stressful situation that occurs when external events or stressors impose demands that cannot be coped with. Therefore, the aim of the present study was to evaluate the sociodemographic factors associated with psychological distress in dental interns from the Peruvian capital facing the COVID-19 pandemic.

**Materials and methods:**

This analytical, observational, cross-sectional study was conducted on 392 Stomatology interns from the Peruvian capital from June to July 2022. The validated COVID-19 Peritraumatic Distress Index (CPDI) scale to detect the levels of psychological distress consisted of four dimensions: negative mood, changes in behavior and cognitive skills, fatigue and hyperreactivity, and somatization. Pearson's chi-square and Fisher's exact test were used for bivariable analysis. In addition, a logit model was used to assess the influence of variables: sex (X1), age group (X2), marital status (X3), monthly economic income (X4), working area in the capital city (X5), and living with people vulnerable to COVID-19 (X6), with the psychological distress levels, considering a significance *p* < 0.05.

**Results:**

The prevalence of psychological distress in dental interns was severe in 6.4% [95% confidence interval (CI): 3.9–8.8%] and mild in 37.8% (95% CI: 33.0–42.6%). According to bivariable analysis, the levels of psychological distress by COVID-19 were not significantly associated with sex (*p* = 0.190), age group (*p* = 0.418), marital status (*p* = 0.554), monthly economic income (*p* = 0.327), working area in the capital city (*p* = 0.993), and living with people vulnerable to COVID-19 (*p* = 0.134). In addition, according to the logistic regression analysis, none of the variables studied was considered an influential factor (*p* > 0.05) in psychological distress presented by dental interns.

**Conclusion:**

The 44.2% of dental interns from the Peruvian capital presented psychological distress facing the COVID-19 pandemic, without any of the possible associated variables of this study significantly affecting this behavioral disorder.

## Introduction

The COVID-19 pandemic severely affected the mental health of populations in general, since in order to control the spread of disease, it was necessary to take sanitary measures such as social distancing, which resulted in isolation and loneliness. Similarly, the economic sector was affected, accentuating the material and economic shortages. It was also necessary to adapt to teleworking, virtual education, and access to medical and psychological care was restricted. In addition to all this, the infodemia by some media contributed to the prevalence of psychological disorders (1, 2).

According to a study conducted during the pandemic of more than 55,000 participants from 40 countries, multiple and wide-ranging vulnerabilities from anxiety to probable depression and suicidal tendencies through distress were recorded (3). The Global Burden of Disease estimated that the COVID-19 pandemic has caused a 27.6% increase in cases associated with major depressive disorder and a 25.6% increase in cases associated with anxiety disorders (4). In Peru, preliminary findings of the population-based survey on mental health during the COVID-19 pandemic, in which 58,349 people participated, showed that 28.5% of all respondents reported depressive symptomatology. Of this group, 41% of respondents reported symptoms associated with mild-to-severe depression and 12.8% reported suicidal ideation. Women and men reported depressive symptomatology in 30.8 and 23.4% of the total cases, respectively. The age group of 18-24 years had the greatest depressive affectation (5).

Psychological distress can be considered a maladaptive response to a stressful situation that occurs when external events or stressors impose demands that cannot be coped with, characterized by a predominance of physical symptoms where there is paralysis of the organism, overwhelm, and a decrease in the person's precision to grasp this phenomenon (6–8). Psychological distress in healthcare is common and may include symptoms of burnout, depression, anxiety, rumination, and perceived stress (9–11). In this context, in recent studies, it has been reported that students, interns, and dental professionals were more likely to develop mood disorders (12–14), for example, Mekhemar et al. (15) reported higher levels of anxiety, stress, and depression in female dentists aged 50 and 59 years old, with immunodeficiency or with a chronic disease and in those who considered the COVID-19 pandemic as a financial risk. In addition, several sociodemographic factors have been reported to be associated with psychological distress in the context of the pandemic and confinement, such as age, gender, marital status, economic income, and cohabitation with vulnerable people, among others (2, 16, 17).

To specifically assess the emotional impact of the COVID-19 pandemic, the *COVID-19 Peritraumatic Distress Index* (CPDI) (18–23) has been designed and consists of four dimensions D1: negative mood, D2: changes in behavior and cognitive skills, D3: fatigue and hyperreactivity, and D4: somatization (19).

The justification of the present study lies in the increase of psychological distress symptoms in students and professionals of health sciences such as medicine, dentistry, and nursing, among others since these are professional areas associated with high academic and clinical workload, which could lead to a decrease in physical and mental capacity, as well as the consumption of harmful substances and development of suicidal tendencies (9, 24, 25). Therefore, the findings of the present study will help to underline the need to include emotional impact management as part of the educational curricula, as well as to develop coping strategies, interventions, and support programs in health, mainly based on stress management in students who have contact with patients, even more so in times of the COVID-19 pandemic (26, 27).

Therefore, the purpose of the present study was to evaluate the sociodemographic factors associated with psychological distress in dental interns in the Peruvian capital facing the COVID-19 pandemic.

## Materials and methods

### Type of study and delimitation

This analytical, prospective, observational, and cross-sectional study was carried out with dental interns from the Peruvian capital from June to July 2022. This manuscript was written according to the STrengthening the Reporting of OBservational studies in Epidemiology (STROBE) guidelines for observational studies (28).

### Sample size and selection of participants

The sample size was 392 dental interns. This was calculated with the Epidat 4.2 statistical program based on a formula for estimating a proportion with an unknown population, considering *p* = 0.5 with a significance level α = 0.05 and a margin of error of 5%. The sampling technique was by a snowball, according to the inclusion and exclusion criteria.

### Inclusion criteria

The inclusion criteria are as follows:

- Dental interns.
- Dental interns residing in the Peruvian capital.
- Dental interns who gave their informed consent and free disposition to be part of the study.
- Dental interns of both sexes with legal age.

### Exclusion criteria

The exclusion criteria are as follows:

- Dental interns who did not complete the entire questionnaire.
- Dental interns who were receiving psychological/psychiatric treatment.

### Variables

The associated factors considered in the study, in relation to the variable psychological distress in the face of COVID-19, were sex (X1), age group (X2) (21, 22), marital status (X3), monthly economic income (X4), working area in the capital city (X5), and living with people vulnerable to COVID-19 (X6) (3).

### Instrument application

The Spanish-validated version for Peru of the COVID-19 Peritraumatic Distress Index Scale (CPDI) was used to detect psychological distress facing the COVID-19 pandemic (19). This scale consisted of 24 items distributed in four dimensions. The first dimension consisted of five items (R1–R5) that assessed negative mood. The second dimension consisted of seven items (R6–R12) that assessed changes in behavior and cognitive skills. The third dimension consisted of seven items (R13–R19) that assessed fatigue and hyperreactivity. The fourth dimension consisted of five items (R20–R24) that assessed somatization. All questions referred to signs and symptoms that dental interns felt in the week prior to the assessment (18, 21). Each item had five ordinal (Likert-type) response alternatives “never,” “occasionally,” “sometimes,” “often,” and “most of the time” with scores from 0 to 4, respectively. Upon answering the full scale, the scores for each item were summed and a total score was obtained, which was converted into a “*COVID-19 Peritraumatic Distress Index” (CPD Index)* based on the total score plus four points (23).

The results of psychological distress levels facing the COVID-19 pandemic were divided into the following ranges (18):

Normal: No psychological distress present (<28 points).

Mild to moderate: Presence of minimal to moderate psychological distress (28–51 points).

Severe: Presence of marked to severe psychological distress (≥52 points).

The instrument's reproducibility test was assessed in a sample of 30 students, and the questionnaire was taken twice at two different times in an interval of 10 days (29), altering the order of the questions to avoid memory bias (test–retest). The results of Pearson's *R* correlation between both scores were very good (*R* = 0.994; CI: 0.985–0.998). The reliability was analyzed with Cronbach's alpha for each dimension, obtaining acceptable values for the first (0.712; CI: 0.664–0.755), second (0.780; CI: 0.745–0.812), third (0.896; CI: 0.879–0.911), and fourth dimensions (0.805; CI: 0.773–0.834). Internal consistency of the entire instrument was very good with a Cronbach's alpha value of 0.932 (CI: 0.922–0.941).

### Procedure

The questionnaire was elaborated in the virtual platform Google Classroom^®^ and was distributed in a self-administered manner *via* a link through the social networks WhatsApp^®^, Facebook^®^, Telegram^®^, and e-mails to different dental interns from seven different universities who were doing their internship in different parts of the Peruvian capital (Central Lima, East Lima, North Lima, and South Lima) with assistance from their hospital internship professors, who were contacted by telephone. Upon clicking on the invitation, the participants were automatically directed to the study aim and the informed consent page with the principal investigator's contact information. Once they agreed to participate, they were directed to the scale (CPD index) with instructions to develop it. However, they were free to decline the assessment if they did not wish to complete it during its course. Personal details such as telephone number, name, and address were not required. The survey was designed to be completed only once. Data were collected and stored in a Microsoft^®^ Excel 2019 spreadsheet, and these data were kept in a password-protected digital folder to which only the researchers had access.

### Statistical analysis

The data were imported by STATA statistical software, version 16.0 (College Station, Texas, USA), and descriptive statistics were used to obtain the absolute and relative frequencies of categorical variables. For bivariable analysis, Pearson's chi-square test and Fisher's exact test were applied for expected values <5. Influencing factors were examined using a logistic regression model (logit model) with an odds ratio (OR). All analyses were carried out considering a significance level of 5% (*p* < 0.05).

### Ethical aspects

All participants gave their virtual informed consent voluntarily. In addition, the present study respected the bioethical principles for medical research on human subjects from the Declaration of Helsinki (30) related to confidentiality, freedom, respect, and non-maleficence since the data were stored in a portable device with a password to which only the researchers had access. This research was approved by the Institutional Ethics Committee of the Universidad Privada San Juan Bautista with resolution No. 823-2022-CIEI-UPSJB.

## Results

Of all dental interns, the female sex was the most predominant with 68.4%. There was also a higher percentage of dental interns between 26 and 35 years of age at 46.7%. Of the total number of dental interns, 83.4% were single. In addition, 58.7% earned <US$250 per month and 76.8% worked as interns on the outskirts of the Peruvian capital. Finally, 54.8% lived with people vulnerable to COVID-19 (Table 1).

**Table 1:** Characterization of sociodemographic variables of dental interns from the Peruvian capital.

**Variable**	**Categories**	**Frequency**	**Percentage**
Sex	Female	268	68.4
	Male	124	31.6
Age group	≤25 years	160	40.8
	26–35 years	183	46.7
	>35 years	49	12.5
Marital Status	Married or cohabiting	65	16.6
	Single	327	83.4
Monthly economic income	≤250 dollars	230	58.7
	250–500 dollars	100	25.5
	500–750 dollars	28	7.1
	>750 dollars	34	8.7
Working area in capital city	Central Lima	91	23.2
	East, North and South Lima	301	76.8
Living with people vulnerable to COVID-19	Yes	215	54.8
	No	177	45.2
**Age**	**Mean**	**Median**	**SD**
	28.5	27.0	6.7

SD, standard deviation.

**Figure 1:**
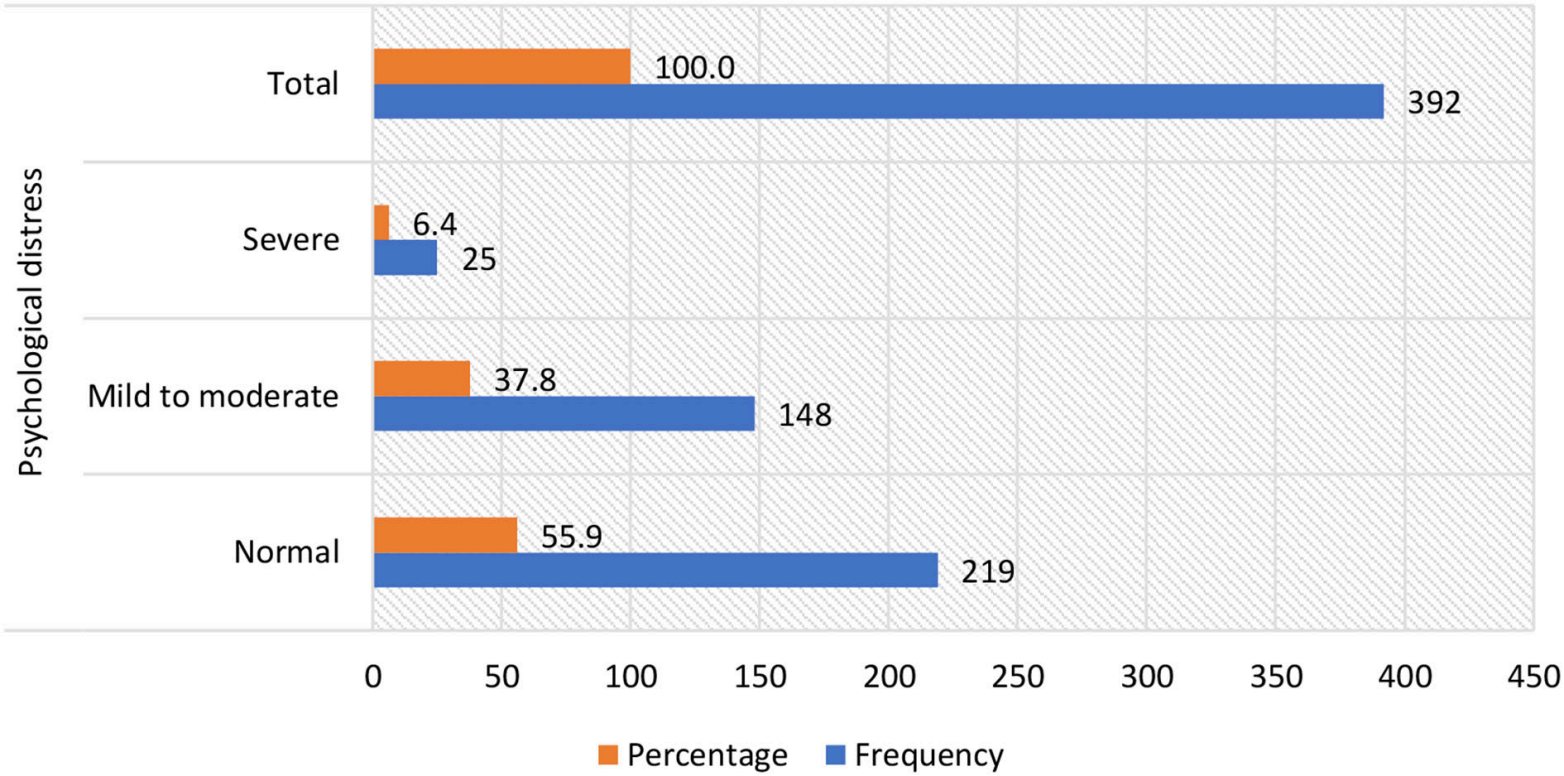
Frequency of dental interns according to their levels of psychological distress.

Of the 392 dental interns surveyed, the prevalence of psychological distress was found to be severe in 6.4% (95% CI: 3.9–8.8%) and mild to moderate in 37.8% (95% CI: 33.0–42.6%). The overall prevalence of psychological distress was 44.2% (95% CI: 39.2–49.1%) (Figure 1).

According to the negative mood of dental interns, the results showed that there was a statistically significant association of sex with R3 (I feel insecure and have been buying a lot of masks, remedies, disinfectant gel, gloves, and/or other household products.) and R4 (I feel compassion for patients with COVID-19 and their families. I feel sad for them.) (*p* < 0.001 and *p* = 0.018, respectively). Age group was significantly associated with R3 and R5 (No matter what I do, I feel empty and helpless.) (*p* = 0.019 and *p* = 0.031, respectively). Marital status was only significantly associated with R3 (*p* = 0.012), while monthly economic income was significantly associated with R2 (I cannot stop imagining that possibly my family or I might be infected and I feel terrified and anxious thinking about it.), R4, and R5 (*p* = 0.003, *p* = 0.039, and *p* < 0.001, respectively). On the other hand, the working area in the capital city was significantly associated with R4 (*p* = 0.029). Finally, living with people vulnerable to COVID-19 was significantly associated with R5 (*p* = 0.045) (Table 2).

**Table 2:** Negative mood associated with sociodemographic factors of dental interns from the Peruvian capital.

**Questions**	**Never**	**Occasionally**	**Sometimes**	**Often**	**Most of the time**	**Sex**	**Age group**	**Marital status**	**Monthly economic income**	**Working area in capital city**	**Living with people vulnerable to COVID-19**
	***f*** **(%)**	***f*** **(%)**	***f*** **(%)**	***f*** **(%)**	***f*** **(%)**	^*^ *p*	^*^ *p*	^*^ *p*	^*^ *p*	^*^ *p*	^*^ *p*
R1. I feel more anxious and nervous than usual	85 (21.7)	109 (27.8)	148 (37.8)	33 (8.4)	17 (4.3)	0.190	0.485	0.317	0.998	0.390	0.809
R2. I can't stop imagining that possibly my family or I might be infected and I feel terrified and anxious thinking about it	120 (30.6)	129 (32.9)	100 (25.5)	28 (7.1)	15 (3.8)	0.140	0.313	0.089	0.003	0.814	0.931
R3. I feel insecure and have been buying a lot of masks, remedies, disinfectant gel, gloves and/or other household products	107 (27.3)	140 (35.7)	93 (23.7)	43 (11.0)	9 (2.3)	< 0.001	0.019	0.012	0.239	0.993	0.199
R4. I feel compassion for COVID-19 patients and their families. I feel sad for them	110 (28.1)	65 (16.6)	97 (24.7)	73 (18.6)	47 (12.0)	0.018	0.152	0.144	0.039	0.029	0.844
R5. No matter what I do, I feel empty and helpless	120 (30.6)	79 (20.2)	103 (26.3)	57 (14.5)	33 (8.4)	0.295	0.031	0.868	< 0.001	0.742	0.045

* Based on Pearson's chi-square, significant association (p < 0.05). In cells where more than 20% of expected values were found to be <5, Fisher's exact test was considered.

According to the changes in behavior and cognitive skills of dental interns, it was observed a statistically significant association of sex with R7 (I am losing faith in people around me.) and R8 (I tend to believe negative news about COVID-19 and have more skeptical opinions about good news.) (*p* = 0.031 and *p* = 0.005, respectively). The age group alone was significantly associated with R7 (*p* = 0.005). Marital status was significantly associated with R9 (I keep an eye on information on COVID-19 all the time. Even if it is not necessary, I cannot avoid it.), R11 (I believe all sources with information about COVID-19 without previously evaluating them.), and R12 (I avoid watching the news about COVID-19 because of the fear it generates me.) (*p* = 0.004, *p* = 0.003 and *p* = 0.045, respectively). Monthly economic income was significantly associated with R8 and R11 (*p* = 0.018 and *p* = 0.036, respectively). On the other hand, working area in the capital city was significantly associated with R6 (I feel powerless and angry with people around me, authorities, and the media) (*p* = 0.007). Finally, living with people vulnerable to COVID-19 was significantly associated with R7 and R11 (*p* = 0.045 and *p* = 0.033, respectively) (Table 3).

**Table 3:** Changes in behavior and cognitive skills associated with sociodemographic factors of dental interns from the Peruvian capital.

**Questions**	**Never**	**Occasionally**	**Sometimes**	**Often**	**Most of the time**	**Sex**	**Age group**	**Marital Status**	**Monthly economic income**	**Working area in capital city**	**Living with people vulnerable to COVID-19**
	***f*** **(%)**	***f*** **(%)**	***f*** **(%)**	***f*** **(%)**	***f*** **(%)**	^*^p	^*^p	^*^p	^*^p	^*^p	^*^p
R6. I feel powerless and angry with people around me, authorities and the media	94 (24.0)	115 (29.3)	100 (25.5)	53 (13.5)	30 (7.7)	0.578	0.109	0.527	0.062	0.007	0.598
R7. I am losing faith in people around me	139 (35.5)	101 (25.8)	97 (24.7)	33 (8.4)	22 (5.6)	0.031	0.005	0.712	0.227	0.538	0.045
R8. I tend to believe negative news about COVID-19 and have a more skeptical opinion about good news	132 (33.7)	133 (33.9)	77 (19.6)	33 (8.4)	17 (4.3)	0.005	0.370	0.960	0.018	0.388	0.379
R9. I keep an eye on information about COVID-19 all the time. Even if it is not necessary. I can't avoid it	148 (37.8)	126 (32.1)	77 (19.6)	25 (6.4)	16 (4.1)	0.995	0.243	0.004	0.144	0.152	0.846
R10. I am constantly sharing news about COVID-19 (mostly negative news)	218 (55.6)	92 (23.5)	58 (14.8)	14 (3.6)	10 (2.6)	0.092	0.126	0.822	0.616	0.578	0.511
R11. I believe all sources with information about COVID-19 without previously evaluating them	232 (59.2)	77 (19.6)	60 (15.3)	16 (4.1)	7 (1.8)	0.245	0.082	0.003	0.036	0.684	0.033
R12. I avoid watching news about COVID-19 because of the fear it generates me	152 (38.8)	102 (26.0)	76 (19.4)	39 (9.9)	23 (5.9)	0.736	0.078	0.049	0.149	0.802	0.431

* Based on Pearson's chi-square, significant association (p < 0.05). In cells where more than 20% of expected values were found to be <5, Fisher's exact test was considered.

According to fatigue and hyperreactivity of dental interns, the statistically significant association of sex was observed with R13 (I feel more irritable and have frequent conflicts with my family.) and R18 (I feel uncomfortable communicating with other people.) (*p* = 0.016 and *p* = 0.008, respectively). Age group was only significantly associated with R14 (I feel tired and sometimes even totally out of strength.), R16 (Because of anxiety, my reactions are slowing down.), R17 (It is difficult for me to make decisions.), and R18 (*p* = 0.016, *p* = 0.032, *p* < 0.001, and *p* = 0.009, respectively). Marital status was only significantly associated with R14 (*p* = 0.034). Monthly economic income was significantly associated with R17 and R19 (I am talking less with my family.) (*p* = 0.038 and *p* = 0.031, respectively). Finally, living with people vulnerable to COVID-19 was significantly associated with R18 (*p* = 0.023) (Table 4).

**Table 4:** Fatigue and hyperreactivity associated with sociodemographic factors of dental interns from the Peruvian capital.

**Questions**	**Never**	**Occasionally**	**Sometimes**	**Often**	**Most of the time**	**Sex**	**Age group**	**Marital status**	**Monthly economic income**	**Working area in capital city**	**Living with people vulnerable to COVID-19**
	***f*** **(%)**	***f*** **(%)**	***f*** **(%)**	***f*** **(%)**	***f*** **(%)**	^*^p	^*^p	^*^p	^*^p	^*^p	^*^p
R13. I feel more irritable and have frequent conflicts with my family	204 (52.0)	98 (25.0)	66 (16.8)	12 (3.1)	12 (3.1)	0.016	0.157	0.116	0.904	0.229	0.287
R14. I feel tired and sometimes even totally out of strength	123 (31.4)	142 (36.2)	90 (23.0)	24 (6.1)	13 (3.3)	0.061	0.016	0.034	0.160	0.347	0.655
R15. It is hard for me to concentrate	138 (35.2)	133 (33.9)	92 (23.5)	17 (4.3)	12 (3.1)	0.110	0.078	0.834	0.271	0.634	0.975
R16. Because of anxiety, my reactions are slowing down	142 (36.2)	108 (27.6)	104 (26.5)	26 (6.6)	12 (3.1)	0.160	0.032	0.629	0.099	0.824	0.903
R17. It is difficult for me to make decisions	119 (30.4)	135 (34.4)	106 (27.0)	26 (6.6)	6 (1.5)	0.232	< 0.001	0.340	0.038	0.699	0.749
R18. I feel uncomfortable communicating with other people	210 (53.6)	100 (25.5)	57 (14.5)	21 (5.4)	4 (1.0)	0.008	0.009	0.640	0.052	0.984	0.023
R19. I am talking less with my family	202 (51.5)	103 (26.3)	59 (15.1)	23 (5.9)	5 (1.3)	0.169	0.232	0.671	0.031	0.305	0.125

* Based on Pearson's chi-square, significant association (p < 0.05). In cells where more than 20% of expected values were found to be <5, Fisher's exact test was considered.

According to the somatization presented by dental interns, a statistically significant association of sex was observed with R20 (During this COVID-19 period, I often feel dizzy or have back pain and/or chest discomfort.) and R24 (I have constipation or frequent urination.) (*p* = 0.001 and *p* = 0.012, respectively). In addition, monthly economic income was significantly associated with R21 (During this COVID-19 period, I often have stomach pain, bloating, and other stomach discomforts.) and R22 (I cannot sleep well. I dream that I or my family are infected with COVID-19.) (*p* = 0.032 and *p* = 0.007, respectively). Finally, living with people vulnerable to COVID-19 was significantly associated with R20 and R22 (*p* = 0.026 and *p* = 0.032, respectively) (Table 5).

**Table 5:** Somatization associated with sociodemographic factors of dental interns from the Peruvian capital.

**Questions**	**Never**	**Occasionally**	**Sometimes**	**Often**	**Most of the time**	**Sex**	**Age group**	**Marital status**	**Monthly economic income**	**Working area in capital city**	**Living with people vulnerable to COVID-19**
	***f (%)***	***f (%)***	***f (%)***	***f (%)***	***f (%)***	^*^p	^*^p	^*^p	^*^p	^*^p	^*^p
R20. During this COVID-19 period, I often feel dizzy or have back pain and/or chest discomfort	172 (43.9)	112 (28.6)	76 (19.4)	29 (7.4)	3 (0.8)	0.001	0.363	0.710	0.187	0.284	0.026
R21. During this COVID-19 period, I often have stomach pain, bloating and other stomach discomfort	183 (46.7)	103 (26.3)	78 (19.9)	22 (5.6)	6 (1.5)	0.446	0.786	0.406	0.032	0.425	0.592
R22. I can't sleep well. I dream that I or my family are infected with COVID-19	280 (71.4)	61 (15.6)	37 (9.4)	9 (2.3)	5 (1.3)	0.623	0.158	0.479	0.007	0.481	0.032
R23. I have lost my appetite	252 (64.3)	79 (20.2)	51 (13.0)	8 (2.0)	2 (0.5)	0.069	0.057	0.824	0.235	0.515	0.253
R24. I have constipation or frequent urination	189 (48.2)	95 (24.2)	70 (17.9)	26 (6.6)	12 (3.1)	0.012	0.151	0.159	0.092	0.425	0.708

* Based on Pearson's chi-square, significant association (p < 0.05). In cells where more than 20% of expected values were found to be <5, Fisher's exact test was considered.

According to bi-variable analysis, the levels of psychological distress of dental interns with more than 2 years of facing the COVID-19 pandemic were not significantly associated with sex (*p* = 0. 190), age group (*p* = 0.418), marital status (*p* = 0.554), monthly economic income (*p* = 0.327), working area in the capital city (*p* = 0.993), and living with people vulnerable to COVID-19 (*p* = 0.134). In addition, according to the logistic regression analysis, none of the variables studied was considered an influential factor (*p* > 0.05) in the psychological distress presented by dental interns (Table 6).

**Table 6:** Psychological distress associated with sociodemographic factors of dental interns in the Peruvian capital.

**Variables**	**Categories**	**Psychological distress**							
		**Bivariable analysis**				**Multivariable analysis**			
		**Normal**	**Mild to moderate**	**Severe**	^*^ *p*	**OR**	**95% CI**		^**^ *p*
		***f*** **(%)**	***f*** **(%)**	***f*** **(%)**			**LL**	**UL**	
**X1: Sex**	Female	145 (37.0)	102 (26.0)	21 (5.4)	0.190	1.39	0.88	2.19	0.154
	Male	74 (18.9)	46 (11.7)	4 (1.0)		Ref.			
**X2: Age group**	≤25 years	87 (22.2)	59 (15.1)	14 (3.6)	0.418	1.13	0.56	2.31	0.731
	26–35 years	104 (26.5)	72 (18.4)	7 (1.8)		1.00	0.51	1.95	0.998
	>35 years	28 (7.1)	17 (4.3)	4 (1.0)		Ref.			
**X3: Marital status**	Married or cohabiting	34 (8.7)	25 (6.4)	6 (1.5)	0.554	1.23	0.69	2.20	0.479
	Single	185 (47.2)	123 (31.4)	19 (4.8)		Ref.			
**X4: Monthly economic income**	≤250 dollars	131 (33.4)	84 (21.4)	15 (3.8)	0.327	0.78	0.36	1.69	0.528
	250–500 dollars	49 (12.5)	45 (11.5)	6 (1.5)		1.13	0.51	2.50	0.766
	500–750 dollars	21 (5.4)	6 (1.5)	1 (0.3)		0.34	0.11	1.03	0.056
	>750 dollars	18 (4.6)	13 (3.3)	3 (0.8)		Ref.			
**X5: Working area in capital city**	Central Lima	51 (13.0)	34 (8.7)	6 (1.5)	0.993	1.04	0.64	1.69	0.866
	East, North and South Lima	168 (42.9)	114 (29.1)	19 (4.8)		Ref.			
**X6: Living with people vulnerable to COVID-19**	Yes	125 (31.9)	81 (20.7)	9 (2.3)	0.134	0.84	0.55	1.27	0.400
	No	94 (24.0)	67 (17.1)	16 (4.1)		Ref.			

* Based on Pearson's chi-square.

** Based on a logit model, p < 0.05 (significant association).

## Discussion

The impact of the disease caused by the SARS-CoV-2 coronavirus affected work patterns, produced temporary unemployment and lack of social interaction, affecting the mental health of populations in general and forcing them to adapt to this new lifestyle. In addition to all this, the fear of contracting the virus and the concern for the health and well-being of close relatives had to be managed (2, 31, 32). All of the above was maximized in health professionals, especially dentists, as they are exposed to known or suspected COVID-19 virus sources during clinical procedures that generate contaminated bioaerosols (24, 33, 34). Therefore, the aim of the present study was to assess the factors associated with psychological distress in dental interns from the Peruvian capital facing the COVID-19 pandemic.

Although several studies (21, 35, 36) reported, under a multivariable logistic regression model, that various factors such as sex, age, and socioeconomic status, among others, influenced psychological distress facing COVID-19, in the present study, there was no significant influence of any of the factors considered. This could be due to the fact that surveys were conducted at a time when there was a low number of infected and deceased persons (June and July 2022), which may have influenced respondents by giving them a false sense of security (37). Similarly, according to Peruvian government regulations, at the time of the survey, all participants had at least three doses of vaccine against COVID-19, which could also have contributed to feeling more confident about infection (38). In addition, it is worth mentioning that when analyzing each item of the CPD Index in each of its dimensions, associations were found with various sociodemographic factors that are relevant to highlight.

It was observed that the sex of dental interns in the Peruvian capital was associated with the feeling of insecurity and the need to buy many masks, remedies, disinfectant gel, gloves, and/or other household products. Similarly, it was associated with compassion and sadness for patients with COVID-19 and their families. It was also associated with the loss of faith in the people around them, relying on negative news about COVID-19 and doubting good news, being irritable, and having frequent conflicts with their family and discomfort when communicating with others. Finally, they were associated with dizziness, back pain or chest discomfort, constipation, or frequent urination. This could be explained according to Di Crosta et al. (39) by the fact that panic buying is influenced by negative emotions such as fear or anxiety, with women being especially more sensitive to particular situations under pressure (40, 41). In addition, trait neuroticism (being anxious and emotionally vulnerable) has been reported to be more common in women (42), as reported by other studies such as Shrestha et al. (43), Hakami et al. (44), and Ali et al. (45) who reported that women had a higher risk of developing psychological distress during the COVID-19 pandemic. Similarly, Sabrina et al. (46) and Gutiérrez et al. (47) indicated that women tend to show greater concern for the safety of their parents and relatives, and the study by Khanagar et al. (25) indicates that female students are more expressive with their emotions, while male students tend to avoid expressing their feelings. On the other hand, in relation to irritability, our findings corroborate with those reported by Zhang et al. (48) who indicated that the general irritability of women is greater than that of men. In addition, García-Sierra et al. (49) found similar results to ours, reporting that the female gender is a risk factor for somatization and psychological distress.

The findings of the present study indicated that the age group of dental interns from the capital city of Peru was associated with the feeling of insecurity and the need to buy many masks, remedies, disinfectant gel, gloves, and/or other household products. It was also associated with the loss of faith in people around them, feelings of tiredness, and diminished strength. Moreover, it was associated with slower reactions due to the anxiety they felt and difficulty in making decisions. Finally, it was associated with discomfort when communicating with other people. This could be supported by Di Crosta et al. (39) and Arafat et al. (50) who reported that under crisis situations that threaten health or disrupt social life, such as that caused by the COVID-19 pandemic, people change their behavior leading them to buy more things than usual (panic buying or compulsive buying). This may manifest with greater intensity in young people, as they are more exposed to digital platforms that disseminate negative information. Similarly, Rens et al. (51) reported that 65% of young people experienced high levels of mental distress in this epidemiological context. Furthermore, according to the Pew Research Center, young adults aged 18–29 years exhibited higher levels of psychological distress than other age groups (52). Also, according to Cook et al. (53), young people, because of the lack of social interaction due to restrictions imposed to control the spread of SARS-CoV-2 coronavirus, were unable to create and preserve bonds of friendship that could provide them a sense of acceptance, trust, security, and support. Similarly, the COVID-19 pandemic is known to have exacerbated the mental health problems of young people in several ways by disrupting college plans, family life, or employment (54).

In the present study, it was found that the marital status of dental interns from the Peruvian capital was also associated with the feeling of insecurity and the need to buy many masks, remedies, disinfectant gel, gloves, and/or other household products. It was also associated with avoiding news about COVID-19 because of the fear it caused them and finally with the feeling of extreme tiredness. This could be supported by Ortega (55) who reported that consumer behavior depends on several factors, including cohabitation. Therefore, those who are single usually live with their parents and/or grandparents who belong to the vulnerable group, thus, in order to protect themselves, they could develop the tendency to buy more personal protection items. Also, Shrestha et al. (43) found that unmarried health professionals had an increased risk of developing a psychological disorder during the COVID-19 pandemic. In addition, the rapid propagation of false information (infodemia) through different digital media led to singles (56), being those who normally have more time to access social networks, being exposed to both real and fictitious news, generating uncertainty about the COVID-19, which is consistent with the findings of Yoshioka et al. (2) who found a significant association of psychological distress with being single or divorced/separated. Similarly, several studies report that loneliness is a risk factor for developing mental disorders under situations of constant pressure (57–60).

On the other hand, the monthly income of stomatology interns in the Peruvian capital was associated with concern for the possible contagion on themselves or their family members, sadness, and compassion for patients with COVID-19 and their families. It was also associated with the feeling of vulnerability and emptiness, with believing negative news and distrusting good news about COVID-19. Finally, it was associated with stomach pain and bloating, and disturbed sleep due to worry about being infected themselves or their family members. These findings could be explained by the fact that, during the pandemic, social attention by government agencies was reduced because healthcare had to be prioritized, affecting those with the lowest income the most (61). Possibly, this situation generated a certain type of psychological distress since they had to go out into the streets to seek sustenance under risky conditions such as coming into contact with infected people, putting their family members at risk of cross-infection. Similarly, our findings are consistent with the study by Sabrina et al. (46) who reported that 58.1% of dental students said that COVID-19 had a negative impact on their financial situation, causing them greater levels of distress. According to Viertiö et al. (58) and Ahnkist et al. (62), financial difficulties in covering household costs appear to have negative effects on mental health. They also reported that change in employment status and an uncertain financial situation was associated with increased psychological distress and fear, which is corroborated by the study of Rahman et al. (63) who found that 51% of participants surveyed had their jobs compromised by the COVID-19 pandemic, suffering from job loss, reduced working hours, and pay cuts.

In the present study, the Peruvian capital area where dental interns worked was associated with feelings of compassion and sadness for patients with COVID-19 and their families, as well as with helplessness and anger with people, authorities, and the media. This could be supported by the study findings of Chen et al. (64) who indicated that peri-urban communities are marked by limited infrastructure, material deprivation, greater insecurity, and lack of health services. This may have an impact on mental health since the infrastructure and work environments may not be a guarantee of protection against contagion as the demand in these areas is higher, being agglomerations unavoidable.

Dental interns from the Peruvian capital who lived with vulnerable persons were associated with feelings of emptiness and fragility, lack of trust in the people around them, trusting sources with information about COVID-19, discomfort when communicating with other people, and also with dizziness, back pain, chest discomfort, and not sleeping well because they were worried about infecting themselves or their family members. This can be explained by what was found in the study by Abdul et al. (65) who reported that relatives of a critically ill or vulnerable patient presented high levels of anxiety, depression, and stress, perhaps due to the fear that these relatives with comorbidities could be severely affected in their health, complicating it to the point of death risk. This concern was heightened during times of pandemic due to the unknown nature of the disease and its treatment, which is consistent with the data on the increase in the COVID-19 impact index associated with the increase in the prevalence of major depressive disorder and anxiety (4).

The importance of the present study lies in the fact that the results obtained could contribute to the assessment of the psychological morbidity of dental interns, in order to establish lines of action and prevent adverse consequences that could lead them to develop emotional, physiological, and behavioral alterations. Similarly, these results will help to plan the necessary modifications in the educational curriculum according to the current context for the design and implementation of strategies and techniques of emotional intelligence as part of it, as well as to provide student support services for unfortunate situations in times of crisis (24).

This study had some limitations, such as not being able to evaluate the respondents in person, because hospitals still restrict access to external personnel so that they can take personalized surveys due to the pandemic. Another factor that has not been considered in this study is having been ill with COVID-19 since many of the interns are young and most of them have been asymptomatic against COVID-19 and 100% of them have had at least three doses of vaccines. It should be noted that this type of study may present potential selection biases since dental interns presented different sociodemographic characteristics, so possible confounding variables such as marital status, monthly economic income, working area of the capital city, and living with people vulnerable to COVID-19 were controlled (21, 35, 36). It should also be recognized that associations of sociodemographic factors with CPD index items found in the present study do not necessarily constitute evidence of causality. Dental interns who received psychological treatment were excluded because their distress could be attenuated by having received support and follow-up by a professional, which would not allow us to have real data about the impact of the pandemic on their emotional state. Also, the present study did not include the evaluation of the academic and clinical load of the dental interns, which could influence the levels of psychological distress, so it is recommended to take them into account for future research. Another limitation was that the snowball sampling method used did not allow for the calculation of a response rate to the invitation made to dental interns. Finally, the cross-sectional design does not allow us to assess the dynamism and sustainability over time of dental interns facing psychological distress.

It is recommended in the future to carry out studies with a longitudinal design that assess the psychological impact of COVID-19 and the level of acceptance of therapeutic support received. Furthermore, it is recommended that the same aim of the present study be pursued with students in preclinical and clinical areas since Hughes et al. (66) reported moderate levels of psychological distress due to students being faced with the abrupt variation of pedagogical learning resources adapted to a virtual environment (67). Additionally, it is suggested that relevant authorities in professional schools and universities take into account the organization of preventive plans and strategies to overcome the long-term effects of psychological distress, involving policymakers, non-governmental organizations, parents, students, and other concerned organizations.

## Conclusion

In summary, considering the limitations of the present cross-sectional study, it can be concluded that 44.2% of the dental interns from the Peruvian capital presented psychological distress facing COVID-19, without any of the possible associated variables in this study significantly influencing this behavioral disorder. However, it is recommended that relevant authorities take into account the organization of preventive plans and strategies in order to manage in a timely manner the technical, economic, pedagogical, psychological, and nutritional assistance of students who undergo hospital internships, to prevent them from developing psychological distress that would seriously affect their work performance and, even worse, their mental health.

## Data availability statement

The raw data supporting the conclusions of this article will be made available by the authors, without undue reservation.

## Ethics statement

This research was approved by the Institutional Ethics Committee of the Universidad Privada San Juan Bautista with resolution No. 823-2022-CIEI-UPSJB. The patients/participants provided their virtual informed consent to participate in this study.

## Author contributions

CC-R conceived the research idea. CC-R, NC-L, ML-C, and GB-V elaborated on the manuscript. MC-M and CC-R collected and tabulated the information. CL-G, NC-L, ML-C, CC-R, and LC-G carried out the bibliographic search. CC-R and AC-P interpreted the statistical results. CC-R, NC-L, GB-V, ML-C, and RA-R helped in the development of the discussion. CC-R, LC-G, RA-R, NC-L, ML-C, and CL-G performed the critical revision of the manuscript. All authors approved the final version of the manuscript.
